# Comparative effectiveness of pneumococcal vaccination with PPV23 and PCV13 in COPD patients over a 5-year follow-up cohort study

**DOI:** 10.1038/s41598-021-95129-w

**Published:** 2021-08-05

**Authors:** Galina L. Ignatova, Sergey N. Avdeev, Vladimir N. Antonov

**Affiliations:** 1 Department of Therapy, South Ural State Medical University, Vorovskogo Str. 66, building 3, Chelyabinsk, 454048 Russian Federation; 2 Sechenov University, Moscow, Russian Federation

**Keywords:** Microbiology, Medical research

## Abstract

Vaccination against *Streptococcus pneumoniae* is among the most effective measures for preventing pneumonia and reducing the rate of chronic obstructive pulmonary disease (COPD) exacerbations. The objective of this work was to evaluate the long-term effectiveness of PCV13 and PPV23 for preventing pneumonia and COPD exacerbations. The open-label, prospective, observational cohort study involved 302 male patients aged ≥ 45 years: PCV13 group (n = 123); PPV23 group (n = 32); and vaccine-naïve group (n = 147). The primary endpoint included the frequency of pneumonia episodes and COPD exacerbations per year over a 5-year follow-up period. The secondary endpoints included the dynamics of dyspnea severity (MMRC), the BODE index, FEV1, the CAT index, the SGRQ score, and the results of 6-min walk test. Vaccination with PCV13 and PPV23 significantly reduces the total rate of pneumonia during the first year after vaccination. Starting with the second year, clinical effectiveness in PPV23 group decreases compared with both PCV13 group and vaccine-naïve patients. Pneumonia by year 5 after vaccination was registered in 47% of patients in the PPV23 group, versus 3.3% of patients in the PCV13 group (*p* < 0.001); COPD exacerbations—in 81.3% versus 23.6%, respectively (*p* < 0.001). Vaccination with PCV13 significantly reduced and maintained the BODE index over the 5-year follow-up period. Although both vaccines have comparable clinical effects during the first year after vaccination, only PCV13 is characterized by persistent clinical effectiveness during the 5-year follow-up period. Patients older than 55 years who received PPV23 have significantly higher risks of having pneumonia episodes more frequently during the long-term follow-up.

## Introduction

Chronic obstructive pulmonary disease (COPD) is a common disorder characterized by ongoing respiratory symptoms and airflow obstruction caused by airway and/or alveolar abnormalities typically emerging after substantial exposure to hazardous particles and gases^1^. Although COPD is preventable and treatable, its periodic exacerbations considerably worsen patients' quality of life and increase the risks of unfavorable outcomes, morbidity, and mortality.

Frequent exacerbations are among the key factors of poor prognosis of COPD in patients without comorbidities^2^. Each new exacerbation of COPD aggravates the disease course. An exacerbation abruptly increases the risks of developing life-threatening complications and irreversible changes in the respiratory system, which reduce the forced expiratory volume (FEV) and worsen clinical symptoms. Influenza and streptococcus pneumonia infections are among the most common reasons for exacerbation; they worsen the overall prognosis of the disease^3,4^. Remaining an active smoker is another significant factor inducing disease exacerbations^5,6^. Smoking cessation improves the disease prognosis and reduces the risks of unfavorable outcomes and frequent exacerbations by 25%. However, in real-life practice, a significant percentage of patients continue smoking in spite of the physician's recommendations. For this very reason, prevention of COPD exacerbations is the key factor for rehabilitation for these patients, since it is associated with improving clinical symptoms, quality of life, as well as patients' physical and emotional status.

The Global Initiative for Chronic Obstructive Lung Disease (GOLD) recommends influenza vaccination at evidence A level due to its ability to reduce hospitalization and mortality rates, and pneumococcal vaccination at evidence B level to prevent community-acquired pneumonia^1^. In this case, the long-term consequences of any of these infections, in particular in elderly patients, may be very serious, up to disability and an increased risk of death. Nevertheless, a specific vaccination protocol, duration, and persistence of the effect depending on patient's characteristics still need to be elucidated for pneumococcal vaccination. Vaccines of two types are currently used to prevent pneumococcal infections: 13-valent conjugate vaccine and 23-valent polysaccharide vaccine. The question regarding the effectiveness and the personalized approach to vaccine selection is rather relevant. On one hand, the 23-valent vaccine with a wide range of serotypes allows one to broaden its spectrum of activity, especially in the regions endemic for these serotypes. On the other hand, the immune responses induced by polyvalent and conjugate vaccines differ fundamentally. Conjugate vaccines proved to have a prolonged effect and persistent clinical effectiveness in all age groups. Furthermore, the long-term effect of using polyvalent vaccines (especially for immunocompromised patients) and the sequelae for the immune system have been insufficiently studied. It was demonstrated for COPD patients that PPV23 significantly reduces the pneumonia incidence rate over a 3-year period in COPD patients younger than 65 years, in patients with severe respiratory disturbance (predicted FEV1 < 40%), and those having cardiovascular comorbidities^4^. However, only the overall effectiveness in reducing the risks of developing pneumonia in elderly patients (above 65 years) and COPD patients has been demonstrated for PCV13^7^. Individual factors responsible for the high or low clinical effectiveness of vaccination for COPD patients have not been studied.

The question regarding the effectiveness of co-immunization with vaccines against *Streptococcus pneumoniae* + influenza virus still remains disputable^8,9^. The use of co-vaccines potentially has a number of social benefits, in particular it simplifies seasonal vaccination protocols. Some studies have demonstrated that upon co-immunization, the positive effect on cardiovascular mortality is more likely to be associated with the influenza vaccine then with the pneumococcal vaccine. However, often, only the polysaccharide pneumococcal vaccine was utilized in these studies.

To ensure personalized selection of the maximally effective vaccination protocol and stratify the risk groups of COPD exacerbation and recurrent pneumonia, it is necessary to compare patients vaccinated using different protocols. The long-term effects should be evaluated not only between the groups, but also with a vaccine-naïve control group having the same risk factors. Therefore, the objective of our study was to compare the effectiveness of vaccination with PPV23 and PCV13 to prevent COPD exacerbation and recurrent pneumonia episodes during a 5-year period after vaccination, as well as to compare the results in the vaccinated groups to the data in the vaccine-naïve control group characterized by the same baseline disease severity and risk factors.

## Methods

### Study design and patient selection

This was a prospective observational cohort study involving patients subjected to routine vaccination between October and December 2012. Male patients diagnosed with COPD (aged ≥ 45 years) followed up at the Chelyabinsk Municipal Center of Pulmonology. COPD was diagnosed according to the GOLD 2011 criteria. All patients were receiving standard treatment according to the GOLD 2011 guidelines^10^. According to the choice of patients, agreed with their physician, and their consent to any type of vaccination, the patients were allocated into three groups: patients vaccinated with the 23-valent vaccine (PPV23 group), those vaccinated with the 13-valent vaccine (PCV13 group), and vaccine-naïve patients (control group, consisted of patients who refused to receive the vaccine). Follow-up duration was 5 years; the key control parameters were evaluated annually. The study inclusion criteria were as follows.

- Male patients aged ≥ 45 years, inclusive.
- Clinically verified diagnosis of COPD made in accordance with the GOLD 2011 guidelines.
- Current or past smokers with ≥ 10 pack-years history of smoking (e.g., at least one cigarette pack per day for 10 years or an equivalent value).
- The patient has undergone spirometry in compliance with the guidelines of the American Thoracic Society (ATS) and European Respiratory Society (ERS) (2005), and reproducible results were obtained.
- The patient is willing and able to attend control visits and undergo all the scheduled analyses and procedures.

#### Exclusion criteria

- The presence of a clinically relevant respiratory comorbid condition along with COPD (e.g., bronchial asthma, tuberculosis, bronchiectasis, or other nonspecific lung diseases).
- Severe comorbid conditions.
- The presence of malignant tumors of any organ (either treated or not) in the medical history over the past 5 years
- Daily oxygen therapy (> 12 h per day).
- Administration of systemic corticosteroids within the past 3 months.

The primary endpoint included the frequency of pneumonia episodes and COPD exacerbations per year over a 5-year follow-up period. The secondary endpoints were chosen to monitor indirect indicators of overall disease severity and disease-associated risks of adverse clinical outcomes and included the dynamics of dyspnea severity (MMRC), the BODE index, FEV1, the CAT index, the SGRQ score, and the results of 6-min walk test.

### Ethics

The study was conducted according to the Good Clinical Practice standards, which ensure that the design, implementation, and communication of data are reliable, that patients’ rights are protected, and that the integrity of subjects is maintained by the confidentiality of their data. The study was approved by the local ethics committee of the Regional Clinical Hospital #4 (protocol 8. 21.10.2012, LIEC of State-Financed Healthcare Institution, Chelyabinsk Regional Clinical Hospital). All patients provided written informed consent in accordance with the Declaration of Helsinki, which included their consent for using their data in analyses and to be presented.

### Data collection

The results of clinical and instrumental analyses (pulse oximetry, spirometry, whole-body plethysmography, and 6-min walk test) were collected at enrollment and then every year. The dyspnea severity was evaluated using the Modified Medical Research Council dyspnea scale (MMRC)^11^ as grade 0–4. Vaccination effectiveness was assessed according to the number of recurrent pneumonia episodes, the number of COPD exacerbations, and the number of hospitalizations because of disease exacerbation.

### Statistical analysis

The calculations were performed using the R Statistical Package (http://www.r-project.org). Descriptive statistics were shown as absolute frequencies or medians with interquartile range. The Mann–Whitney U-test, or ANOVA, or Pearson’s χ^2^ test, or Fisher's exact test and non-parametric Kruskal–Wallis test by rank and median multiple comparisons were used depending on type of the analyzed data.

All the reported *p* values were based on two-tailed tests of significance; the *p* values < 0.05 were regarded as statistically significant. The STATISTICA 7.0 software (StatSoft, USA) and RStudio software version 1.0.136 (Free Software Foundation, Inc., USA) with R packages version 3.3.1 (R Foundation for Statistical Computing, Austria) were used for the analyses.

### Ethics approval and consent to participate

The study was approved by the local ethics committee of the Regional Clinical Hospital #4 (protocol 8. 21.10.2012) (Chelyabinsk, Russia).

## Results

### Baseline characteristics

Over the period between October and December 2012, 150 patients were vaccinated with PCV13; 32 patients, with PPV23. The study included a control group consisting of 212 vaccine-naïve patients. After adjustment in relation to age, dyspnea severity, and duration of COPD, 123 patients vaccinated with PCV13, 32 patients vaccinated with PPV23, and 147 patients who had not obtained vaccination for various reasons were enrolled. Table 1 summarizes the overall characteristics of patients at baseline.

**Table 1:** Baseline demographic and clinical characteristics of patients enrolled in the study.

Parameter	No vaccination (n = 147)	PPV23 (n = 32)	PCV13 (n = 123)	*p* value (Kruskal–Wallis test)
**Demography and anamnesis**				
Age (years)	63 (60:66)	60 (55:64.25)	61 (57:66)	0.133
Aged > 65 years	56 [38.1%]	8 [25%]	36 [29.27%]	0.181
Age of COPD onset	6 (4:7)	6 (4:6.25)	5 (2:8)	0.103
BMI (at baseline)	21 (19:23)	21 (19:23.25)	21 (19:23)	0.387
**COPD characteristics**				
CAT (at baseline)	25 (21.5:30)	24 (21.8:30.3)	25 (21:30.5)	0.924
MMRC				
Grade 2	19 [12.93%]	6 [18.75%]	12 [9.76%]	0.219
Grade 3	76 [51.7%]	18 [56.25%]	56 [45.53%]	
Grade 4	52 [35.37%]	8 [25%]	55 [44.72%]	
BODE index	5 (4:6)	5 (4:5.25)	5 (4:6)	0.193
6MWT (at baseline)	370 (318:456)	390 (313:497)	370 (340:425)	0.453
SGRQ	39 (35:47)	37.5 (33:47)	38 (35:46)	0.477
Exacerbations over the year preceding vaccination				
None	24 [16.33%]	7 [21.88%]	22 [17.89%]	0.619
One exacerbation	122 [82.99%]	25 [78.12%]	98 [79.67%]	
Two exacerbations	1 [0.68%]	0 [0%]	3 [2.44%]	
Hospitalizations (for the exacerbation or routine) over the year preceding vaccination				
None	13 [8.84%]	4 [12.5%]	10 [8.13%]	0.625
One hospitalization	133 [90.48%]	28 [87.5%]	110 [89.43%]	
Two hospitalizations	1 [0.68%]	0 [0%]	3 [2.44%]	
**Pneumonia episodes**				
Pneumonia episodes over the year preceding vaccination, total				
None	120 [81.63%]	24 [75%]	104 [84.55%]	0.492
One pneumonia	27 [18.37%]	8 [25%]	18 [14.63%]	
Two pneumonias	0 [0%]	0 [0%]	1 [0.81%]	
Pneumonia episodes requiring hospitalization over the year preceding vaccination				
None	120 [81.63%]	27 [84.38%]	104 [84.55%]	0.715
One hospitalization with pneumonia	27 [18.37%]	5 [15.62%]	18 [14.63%]	
Two hospitalizations with pneumonia	0 [0%]	0 [0%]	1 [0.81%]	

Continuous numeric values are shown as Median (interquantile range); categorical values are shown as absolute numbers and percentage of group size.

*COPD* chronic obstructive pulmonary disease, *BMI* body mass index, *CAT* COPD assessment test, *MMRC* Modified Medical Research Council dyspnea scale, *6MWT* 6-min walk test, *SGRQ* St. George’s Respiratory Questionnaire.

The patients were matched for age, duration of COPD, and severity of clinical signs of COPD. After the adjustment, each group contained patients with MMRC ≥ 2. Most of the enrolled patients (82.5%) had at least one COPD exacerbation during the year preceding the enrollment. At least one pneumonia episode was reported by 17.9% of patients during the preceding year.

### Effectiveness of vaccination in preventing pneumonia

**Figure 1:**
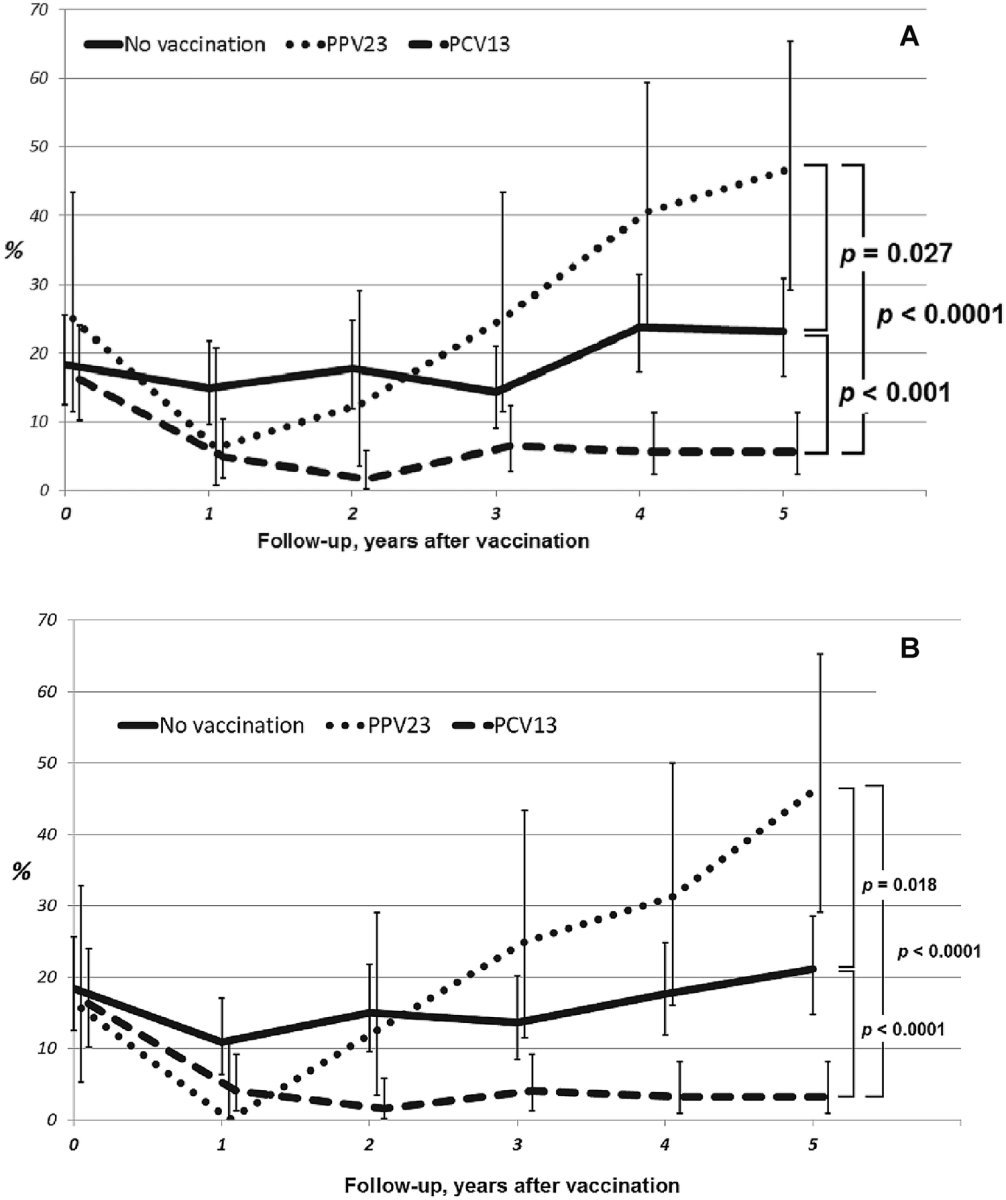
Dynamics of total pneumonia rate (**A**) and rate of hospitalizations with pneumonia (**B**) in the study groups. p value showed ANOVA statistics between groups. The p value shows ANOVA statistics between groups.

Each patient was followed up for 5 years after the baseline characteristics had been collected. The pneumonia rate was comparable at baseline and significantly decreased in both vaccinated groups compared to vaccine-naïve patients (Fig. 1). The percentage of patients having at least one pneumonia episode reduced to 4.9% in the PCV13 group and to 6.3% in the PPV23 group versus 15% level in the vaccine-naïve patients (*p* = 0.024 and *p* = 0.707, respectively). However, patients in the PPV23 group showed significantly worse results during the second year after the vaccination even compared to those in the control (vaccine-naïve) group, and reached a pneumonia rate of 47% by year 5 after the vaccination (as compared with 23.1% in the control group without vaccination, p = 0.027).

### Effectiveness of vaccination for reducing the rate of COPD exacerbations

**Figure 2:**
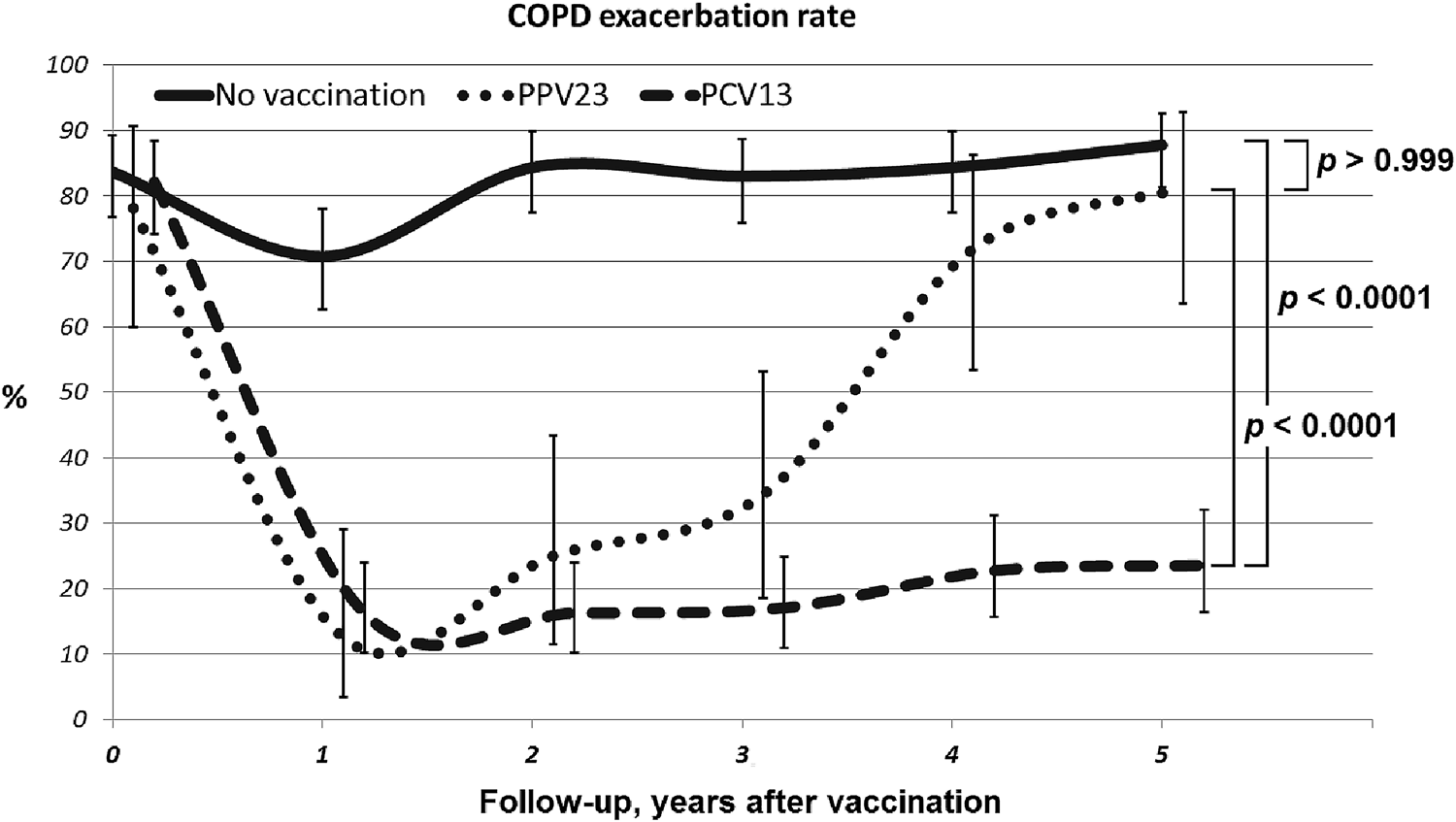
Dynamics of the COPD exacerbation rate in the different study groups. The p value shows ANOVA statistics between groups.

Both types of vaccines were strongly associated with significant reducing of COPD exacerbation rate as compared to the control group (Fig. 2). However, in the PPV23 group, this effect was gradually declining after year 1 post-vaccination and reached the level comparable to that for vaccine-naïve patients by year 5 post-vaccination.

**Figure 3:**
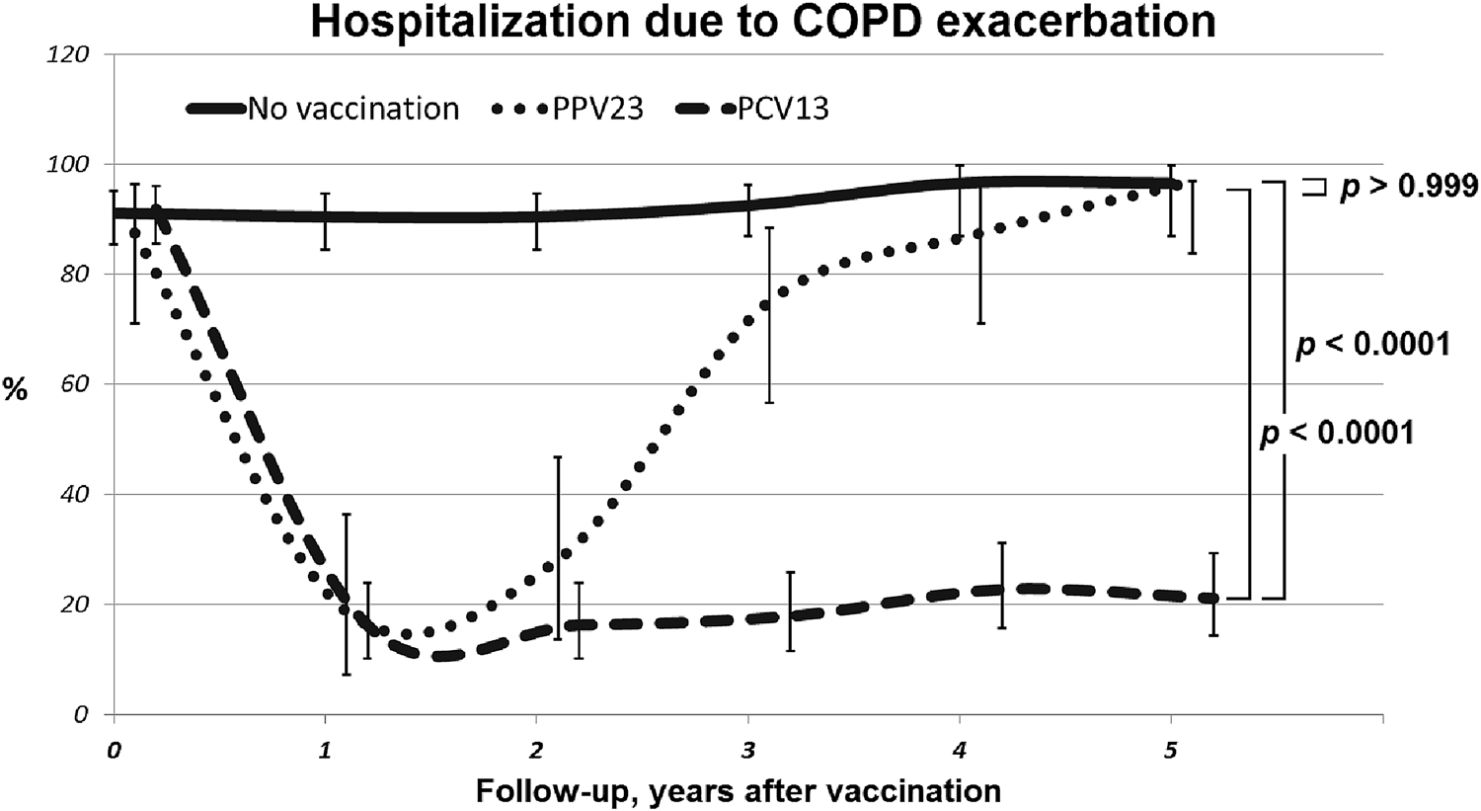
Dynamics of hospitalizations due to COPD exacerbation in the study groups. The P value shows ANOVA statistics between groups.

Similar dynamics were observed for the hospitalization frequency (Fig. 3). At baseline, the overall percentage of patients having at least one hospitalization per year was 90.2%. Vaccination had a significant effect during the first year of the follow-up: only 18.8% of PPV23 patients and 16.3% of PCV13 patients were hospitalized. In the PCV13 group, this effect persisted during the entire follow-up period, and the percentage of patients having disease exacerbation over the 5-year period was ≤ 23%. In the PPV23 group, this effect was significantly reduced after the 2-year follow-up: the exacerbation rate was 75, 88, and 97% during the third, fourth, and fifth years of the follow-up, respectively.

### Effectiveness of vaccination for improving patients' quality of life and respiratory function parameters

**Figure 4:**
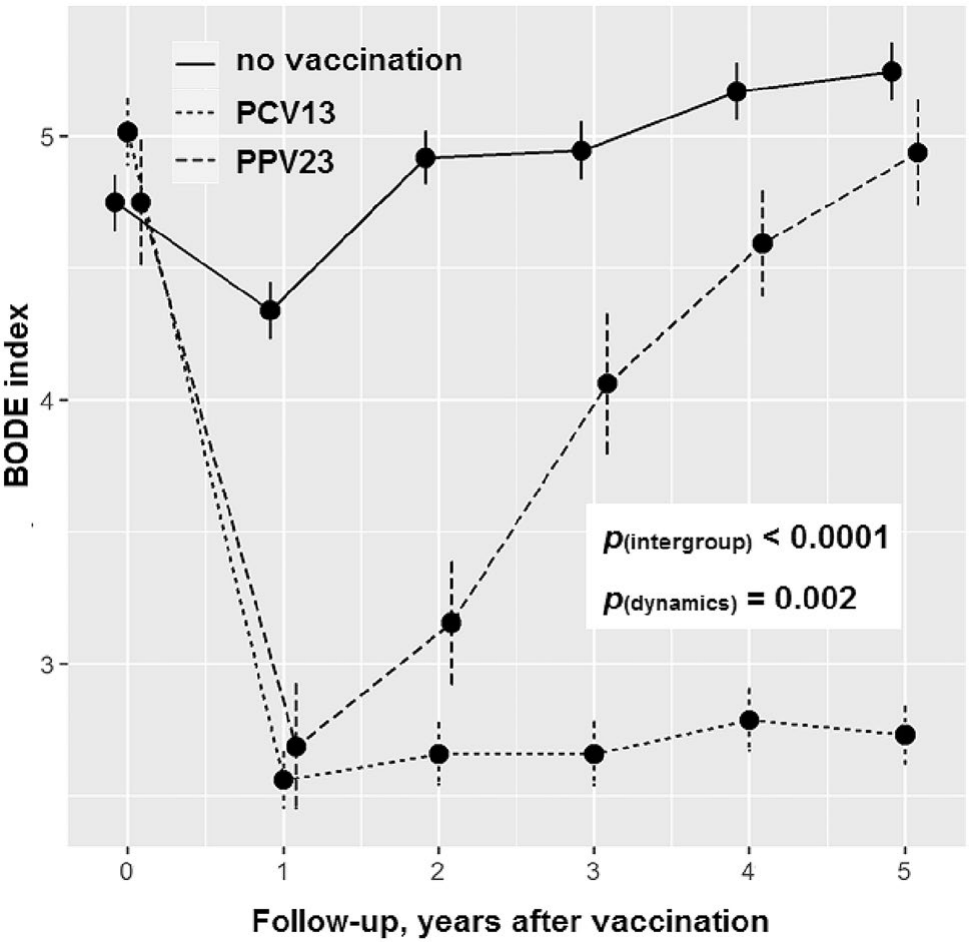
Dynamics of BODE index in study groups during follow-up period. The p value shows ANOVA statistics between groups.

Assessment of the comprehensive effect of vaccination or the absence of vaccination on the BODE index (a multidimensional grading system for evaluating the health status of patients and for predicting mortality risk) index^12^ demonstrated that although both utilized vaccines significantly reduced the mortality rate during the first 2 years after vaccination, but only the PCV13 vaccine provided a persistent effect for 3 years or longer (Fig. 4). The BODE score was reduced by one or more points 1 year after the vaccination in 30 (93.8%) PPV23 patients, 123 (100%) PCV13 patients, and only 75 (51%) vaccine-naïve patients (*p* < 0.0001). However, the BODE score increased significantly in the PPV23 group in the following 4 years. At the end of follow-up period its median was 5 (IQR 4:5) in the PPV23 group and 5 (4:6) in the no vaccination group (p > 0.999). In the PCV13 group the BODE score remained low with 3 (2:3) points after 5 years (p < 0.0001 as compared with both the PPV23 group and the no vaccination group).

**Figure 5:**
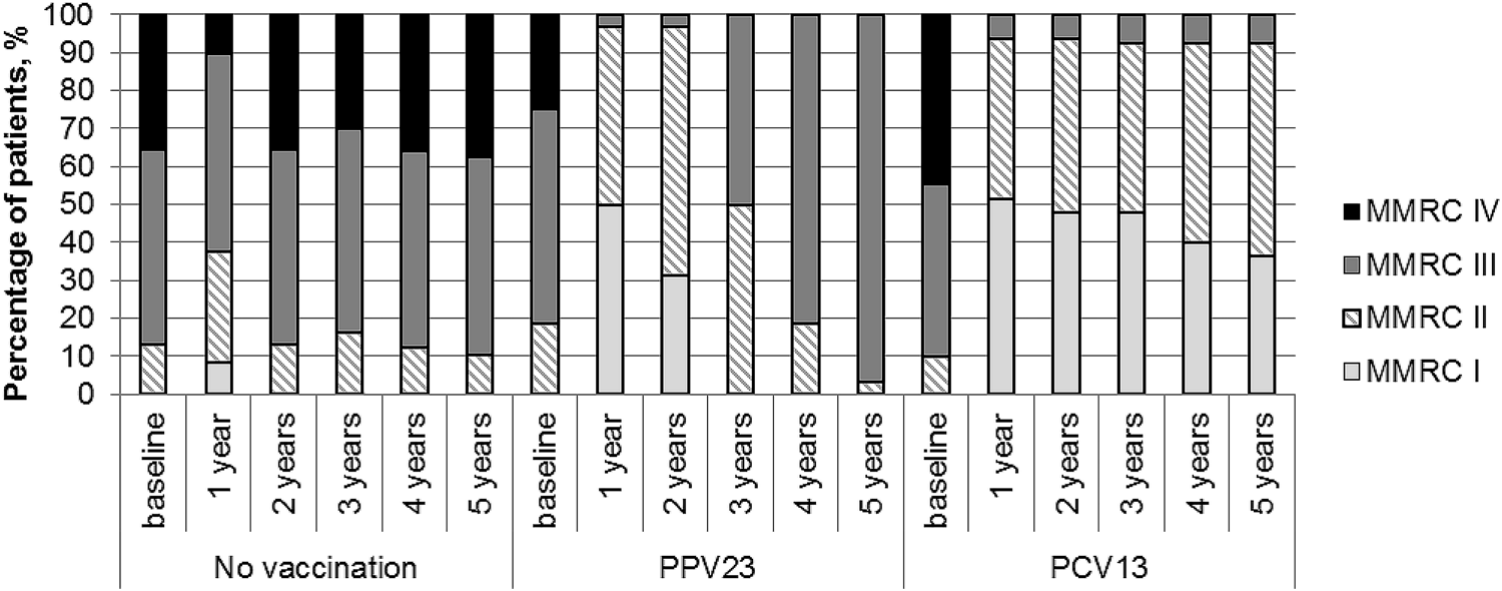
The dynamics of dyspnea severity (MMRC) in the study groups.

Dyspnea severity by MMRC in patients was monitored during the entire follow-up period (Fig. 5). At baseline, the groups were comparable in terms of MMRC (*p* = 0.219); patients having grade 3 predominated in all the groups (52% in the vaccine-naïve group; 56% in the PPV23 group; and 46% in the PCV13 group). A smaller percentage of patients had grade 4, and the lowest number of patients had grade 2. However, as early as 1 year after vaccination, the groups significantly differed in terms of dyspnea severity. In all vaccinated patients (even those who had grade 4 dyspnea at baseline), MMRC decreased to grade 3 or even grade 2, and this effect persisted throughout the entire follow-up period. Even in the vaccine-naïve group, the percentage of patients having grade 4 dyspnea decreased during the first year after vaccination. The overall improvement in health status of all patients during the first year could have resulted from the fact that patients were visiting the physician on a regular basis and, therefore, were more compliant to the therapy received. Nevertheless, this trend towards improvement was maintained only in the PCV13 group. Moreover, the respiratory function parameters were worsened in the PPV23 group down to a level lower than the baseline level: none of the patients had grade 1 by the end of the follow-up period, while grade 2 was observed in only one (3.1%) patient.

**Figure 6:**
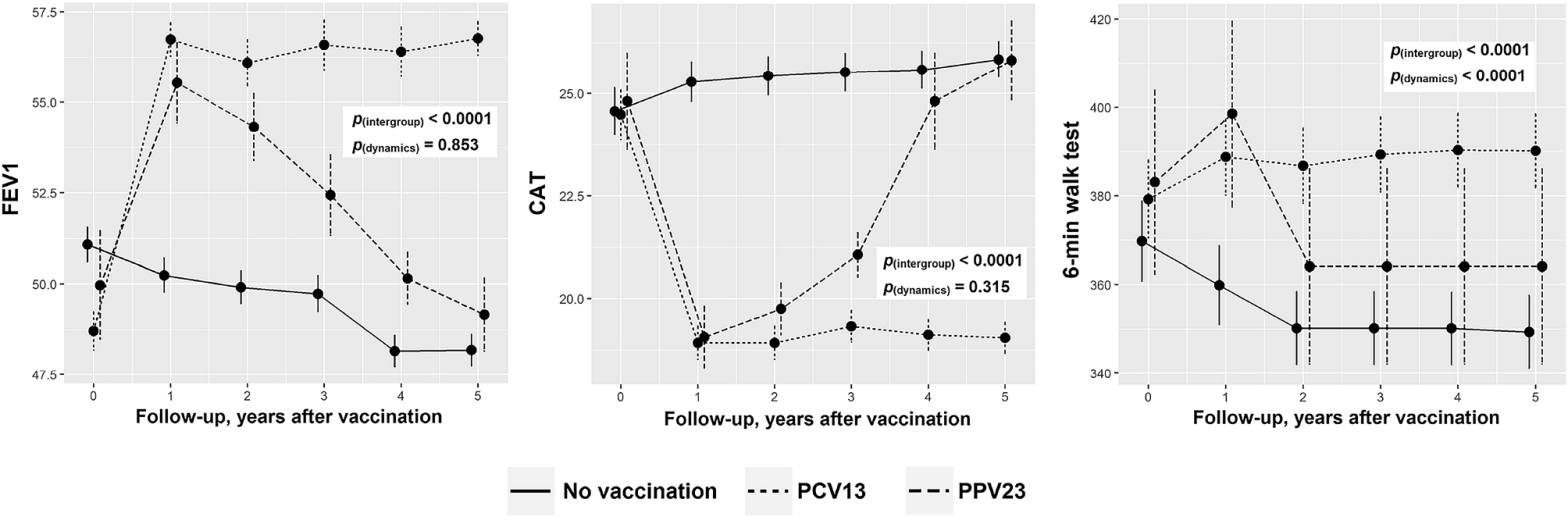
Dynamics of FEV1, CAT, and 6-min walk test in the study groups. The p value shows ANOVA statistics between groups.

Assessment of the dynamics of other clinical and laboratory signs showed that there was an overall trend towards improvement of the health status of vaccinated patients compared to vaccine-naïve ones during the first year in terms of such parameters as FEV1, CAT, and 6-min walk test results (Fig. 6). During the following 4 years, the clinical effect persisted only in the PCV13 group, while the condition of the patients was significantly worsened in the PPV23 group, and parameters comparable to those in the vaccine-naïve group were reached by year 5.

### Factors associated with favorable clinical outcomes

**Figure 7:**
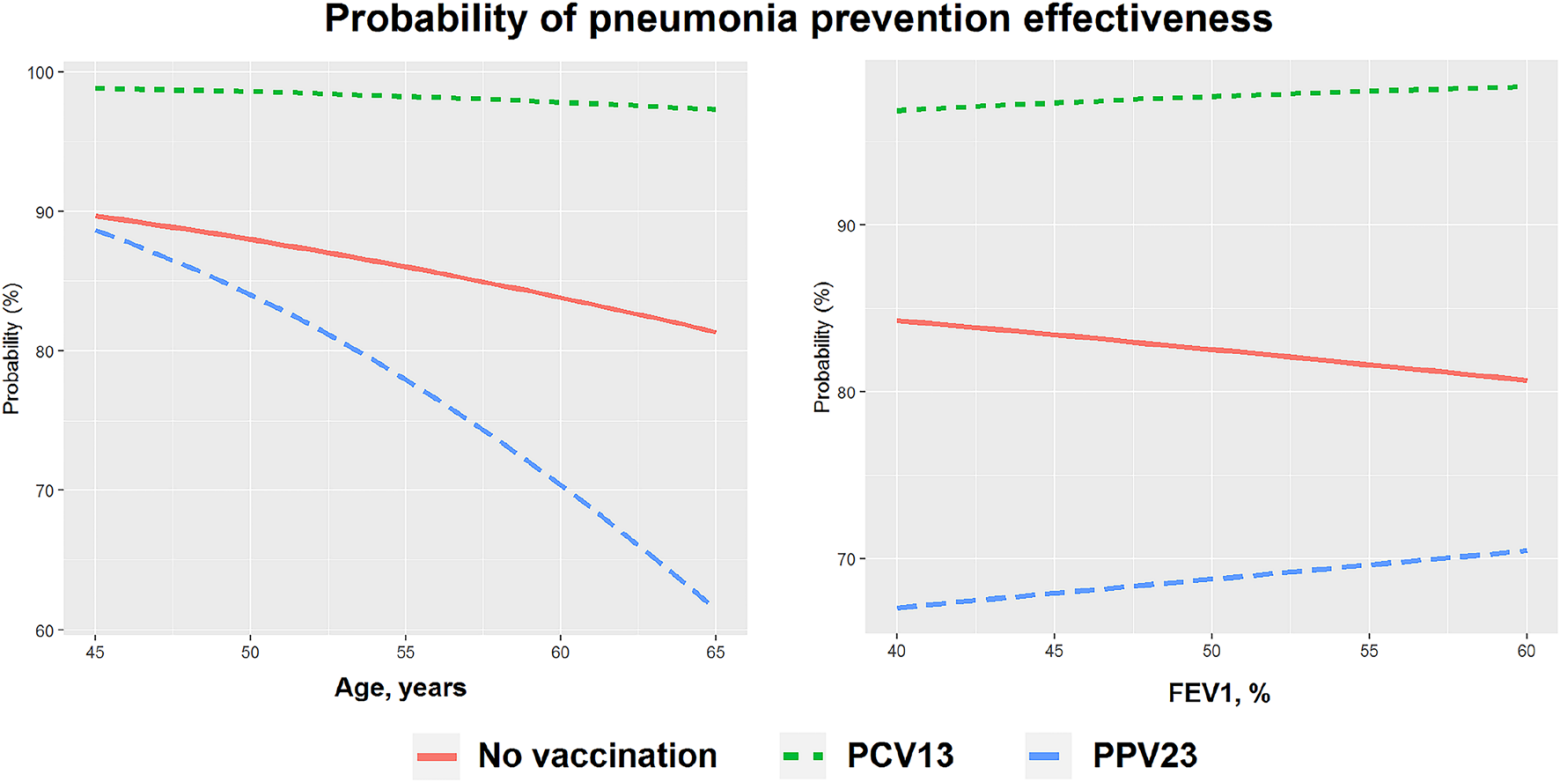
Multivariable model of probability of pneumonia prevention effectiveness.

Assessing the likelihood of reduced rate of pneumonia episodes (or their absence) showed that two main factors associated with favorable outcomes included vaccination with PCV13 and age at vaccination (Fig. 7). The vaccine type was a much more important factor compared to the baseline FEV1, which had no substantial effect on the patient's prognosis. An analysis of the risk model showed that regardless of the age of the patient or FEV1 at the moment of vaccination, PCV13 could significantly reduce the frequency of pneumonia or completely prevent it in more than 95% of patients for up to a 5 year period. At the same time, the use of PPV23 is effective only for patients younger than 55 years and has a limitation on the duration of the effect.

## Discussion

Comparison of clinical effectiveness of vaccines for preventing pneumonia, as well as their mediated effect on the health status of COPD patients, demonstrated that both PCV13 and PPV23 are effective during the first 1–2 years after the vaccination. However, the effectiveness of PPV23 decreases noticeably over time: PPV23 ensured proper protection only within 2 years after vaccination, while PCV13 exhibited a high level of protection during the entire follow-up. Furthermore, the risk of complications in patients vaccinated with PPV23 increased after 3 years after the vaccination compared to vaccine-naïve patients.

Vaccination effectiveness directly depends on various factors, such as the serotype pattern, patient age, and the health status at baseline. Many researchers have mentioned the positive effect of vaccination on outcome^13^ and the cost effectiveness of vaccination^14^ for PPV23 and PCV13^7^. However, for most studies where the vaccines were compared directly, the follow-up period was limited (3 years after the vaccination). This fact does not allow one to take into account the effect of the decline in effectiveness of vaccination with PPV23 and PCV13 over time. Hence, the question regarding the long-term vaccination effectiveness still remains open. This question becomes especially relevant in the presence of risk factors, such as smoking, FEV1^15^, cardiovascular pathology^16^, and other factors^12,17^ worsening the prognosis for developing severe forms of pneumonia. In the current study, all patients had high risk factors for developing severe pneumonia: age > 45 years; smoking; and dyspnea severity grade assessed using the MMRC scale ≥ 2. Nevertheless, pneumococcal vaccination was shown to effectively prevent pneumonia in these patients.

The previous studies assessing the effectiveness of PPV23 showed that the patients differed in terms of clinical effectiveness of vaccination depending on their age^4^. The risk of vaccine failure increased significantly in patients older than 65 years. Our study has confirmed this observation and demonstrated that this effect is valid only for the polysaccharide vaccine. For PCV13, patient age was not a significant factor affecting vaccine effectiveness. Our study found no evidence for the earlier demonstrated influence of FEV1 on vaccine effectiveness. This was probably because only 2.9% of patients in our sample had FEV < 40%, which was regarded as the threshold level for this parameter.

Although the primary objective of vaccination is to reduce the risk of pneumonia in patients with COPD, it also indirectly affects the patient's overall health status and risks associated with adverse clinical outcomes. The study^7^ demonstrated that vaccination with PCV13 reduced the risk of hospitalization (for any reason) in stable patients having no exacerbations (non-exacerbators), but not in patients had having COPD exacerbations within the previous year. In our study, we found that vaccination with PCV13 was associated with a significant decline in exacerbation and hospitalization rates. Before enrollment, the rate of hospitalization because of COPD exacerbation was > 90%. Administration of any pneumococcal vaccine reduced the exacerbation rate over fourfold during the first year. Hence, pneumococcal vaccination has a positive effect on the course of COPD, which includes reduced exacerbation rate and improved overall quality of life. However, reduced hospitalization rates were stably observed only for PCV13, while the exacerbation rate and the rate of exacerbation-related hospitalizations increased abruptly as early as 2 years after vaccination with PPV23. We also demonstrated that vaccinated patients had improvements in the BODE index, MMRC, and other indicators of disease severity 1 year or more after vaccination. Although there is no direct relationship between vaccination and BODE index, an indicator of COPD-related mortality, or MMRC, a dyspnea score, there may be an indirect association with both a significant decrease in the rate of severe infections and disease exacerbations in these groups (which may have lead to partial improvement in lung function and exercise tolerance) and the fact that treatment compliance of the patients may have been additionally stimulated by the attending physician during hospitalization.

The observed increase in the percentage of patients with severe pneumonia in the PPV23 group after 3-year follow-up may potentially be related to depletion of memory B-cell pool. Polysaccharide vaccines stimulate a short-lived B-cell response as they influence the terminal differentiation of the existing memory B-cells into plasma cells, thus depleting the memory B-cell pool^18^. This process may cause an overall decline in immune response. In combination with the elderly age and the numerous risk factors in COPD patients, this process may increase pneumonia severity and the frequency of pneumonia episodes in patients 3–5 years after vaccination with PPV23 even compared to vaccine-naïve patients.

Our study has a number of limitations. First, it has a non-randomized design due to ethical considerations: only patients having additional administrative reasons and warned about the potential risks were enrolled to the vaccine-naïve groups. In order to minimize the risk of bias associated with the non-randomized study design, we employed PSM methods to select matching patient groups. Second, size of the patient groups differed significantly. Third, the study involved PP analysis rather than ITT. Nevertheless, this study was the first study to analyze long-term (5-year) clinical effectiveness of vaccination with PCV13 and PPV23 in patients with COPD. Further trials involving large cohorts and registries will make it possible to assess additional risks associated with the long-term effect of hyporesponsiveness in patients after vaccination with PPV23 and its potential effect on quality of life and the risks of unfavorable outcomes.

## Conclusion

Pneumococcal vaccination with PCV13 and PPV23 significantly improves the clinical status of COPD patients: there are positive trends for parameters like the dyspnea rate, exercise tolerance, reduction of the rate of pneumonia episodes and exacerbations of the underlying disease. However, while the positive effect persists over a 5-year follow-up for PCV13, it gradually declines in patients vaccinated with PPV23 starting at year 2 after the vaccination. Vaccination with PPV23 was associated with a higher risk of severe pneumonia 3 years post-vaccination and further on compared to the risk in vaccine-naïve patients.

## Data Availability

Datasets generated during and/or analysed during the current study are available from the corresponding author on reasonable request.

## Abbreviations

COPD: Chronic obstructive pulmonary disease
FEV: Forced expiratory volume
GOLD: Global initiative for Chronic Obstructive Lung Disease
ATS: American Thoracic Society
ERS: European Respiratory Society
MMRC: Modified Medical Research Council
